# Lipocalin 2 as a potential liquid biopsy marker for early detection of bladder cancer

**DOI:** 10.1002/ctm2.70540

**Published:** 2025-12-09

**Authors:** Mi‐So Jeong, Jeong‐Yeon Mun, Gi‐Eun Yang, Seung‐Woo Baek, Sang‐Yeop Lee, Sung Ho Yun, Seung Il Kim, Jae‐Jun Kim, Seo‐Yeong Yoon, Jong‐Kil Nam, Yung‐Hyun Choi, Hyeok Jun Goh, Tae‐Nam Kim, Sun‐Hee Leem

**Affiliations:** ^1^ Department of Biomedical Sciences Dong‐A University Busan South Korea; ^2^ Research Center Dongnam Institute of Radiological & Medical Sciences Busan South Korea; ^3^ Department of Pathology and Cell Biology Columbia University Medical Center New York New York USA; ^4^ Department of Health Sciences The Graduated of Dong‐A University Busan South Korea; ^5^ Genomic Medicine Research Center Korea Research Institute of Bioscience and Biotechnology Daejeon South Korea; ^6^ Research Center for Bioconvergence Analysis Korea Basic Science Institute Ochang South Korea; ^7^ Center for Research Equipment Korea Basic Science Institute Ochang South Korea; ^8^ Department of Urology Pusan National University Yangsan Hospital Pusan National University School of Medicine, Research Institute for Convergence of Biomedical Science and Technology Yangsan South Korea; ^9^ Department of Biochemistry College of Oriental Medicine Anti‐Aging Research Center Dong‐eui University Busan South Korea; ^10^ Department of Urology Dong‐A University College of Medicine Busan South Korea; ^11^ Department of Urology Pusan National University Hospital Pusan National University School of Medicine, Biomedical Research Institute and Pusan National University Hospital Busan South Korea

1

Dear Editor,

Research on liquid biopsy markers is actively ongoing as an alternative diagnostic method for patients with bladder cancer (BC), which frequently recurs.^1^, ^2^, ^3^ Our study shows that LCN2 expression is linked to BC progression and may serve as a valuable urinary biomarker for identifying early‐stage patients and predicting outcomes.

To identify secreted proteins linked to BC progression, we analysed stepwise 5637 gemcitabine‐resistant cell (GRC) lines established in our previous study.^4^ The conditioned media (CM) from highly motile GRC sublines enhanced invasion and migration (Figure S1A,B). Liquid Chromatography‐Tandem Mass Spectrometry (LC‒MS/MS) analysis of concentrated CM revealed 408 differentially expressed proteins, with 178 upregulated in the highly mobile P7 cell line. Ingenuity pathway analysis identified 56 proteins associated with three motility‐related pathways, 17 of which overlapped between expression and pathway analyses (Figure 1A). Notably, LCN2 demonstrated the strongest differential expression in RNA sequencing data, prompting further investigation due to its established correlation with motility.^4^ LCN2 has been associated with tumour progression and metastasis and has been proposed as a non‐invasive prognostic indicator,^5^, ^6^, ^7^, ^8^ although its precise role in BC remains unclear.

**FIGURE 1:**
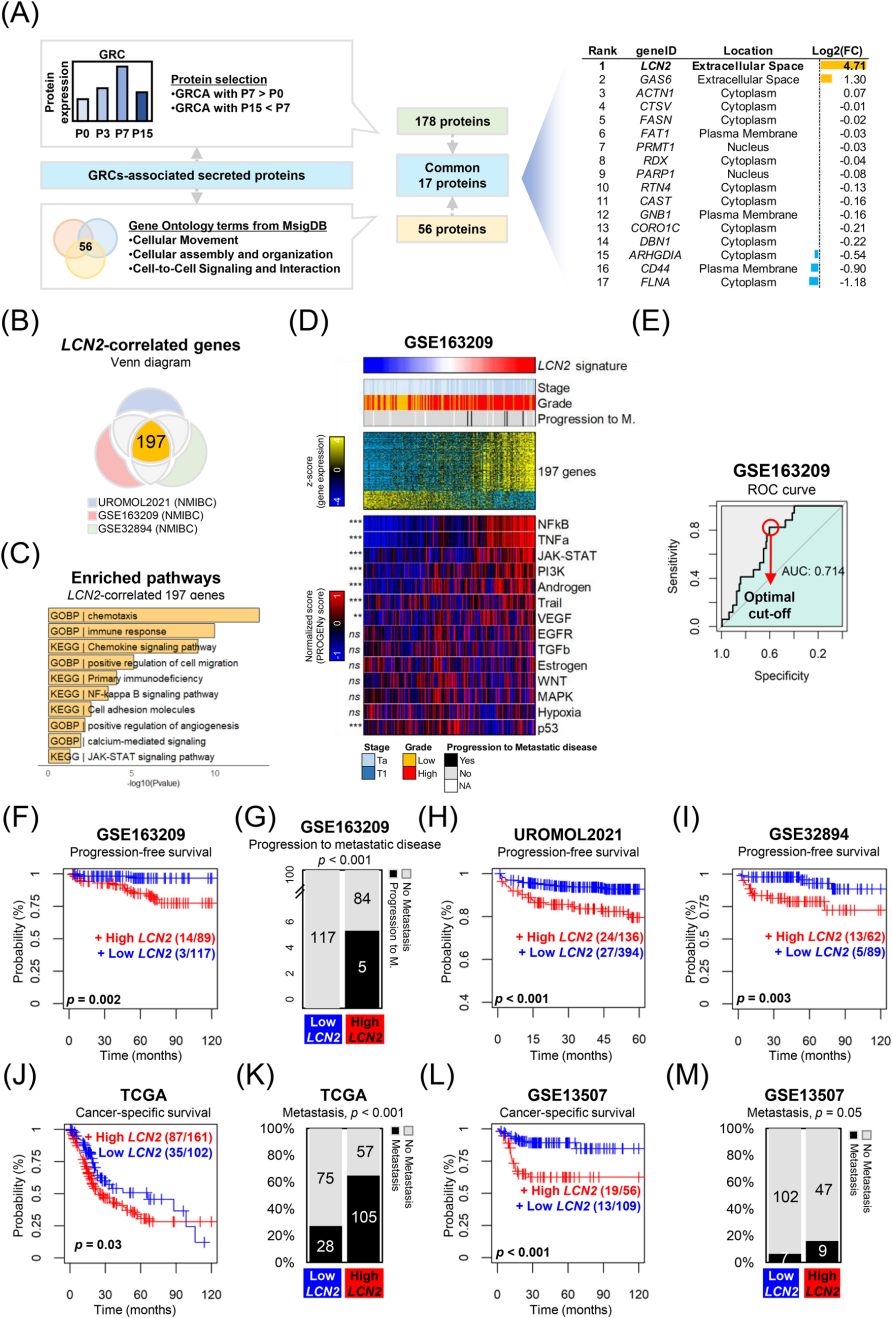
Identification of LCN2 as a metastatic biomarker and its prognostic association with bladder cancer progression. (A) Workflow for selecting LCN2 from gemcitabine‐resistant cells (GRCs) through secretome and RNA sequencing analysis. (B) Venn diagram illustrating the overlap of LCN2‐correlated genes identified across three independent datasets (UROMOL2021, GSE163209 and GSE32894). (C) Enriched biological pathways associated with the 197 LCN2‐correlated genes. (D) Heatmap displaying the expression patterns of the 197 LCN2‐correlated genes in the GSE163209 dataset. Gene expression (*z*‐score) is categorised by tumour stage (Ta/T1), grade (low/high) and progression to metastatic disease. (E) GSE163209 cohort was classified based on area under the receiver operating characteristic (ROC) curve (AUC) values derived from ROC analysis with progression‐free survival. (F) Kaplan‒Meier curve depicting progression‐free survival in the GSE163209 dataset. (G) Bar plot illustrating the proportion of patients who progressed to metastatic disease in the GSE163209 dataset. (H) Kaplan‒Meier curve showing progression‐free survival in the UROMOL2021 dataset. (I) Kaplan‒Meier curve indicating progression‐free survival in the GSE32894 dataset. (J) Kaplan‒Meier curve demonstrating cancer‐specific survival in the TCGA bladder cancer cohort. (K) Bar plot representing the proportion of patients who developed metastases in the TCGA cohort. (L) Kaplan‒Meier curve illustrating cancer‐specific survival in the GSE13507 dataset. (M) Bar plot showing the proportion of patients who developed metastases in the GSE13507 dataset.

The GSE13057 dataset confirmed elevated LCN2 expression in BC tissues compared to normal tissues (Figure S1C). In the 5637GRC model, both intracellular levels and secretion of LCN2 increased at P3 and P7 stages, which were characterised by higher motility (Figure S1D‒H). Analysis of three NMIBC datasets (UROMOL2021, GSE163209 and GSE32894) revealed that 197 genes consistently correlated with LCN2 expression (Figure 1B). Pathway enrichment analysis linked these genes to biological processes involved in cell motility (Figure 1C). High LCN2 expression was associated with activation of Nuclear Factor kappa‐light‐chain‐enhancer of activated B cells (NF‐κB), Janus Kinase‐Signal Transducer and Activator of Transcription (JAK‒STAT) and Phosphoinositide 3‐Kinase (PI3K) signalling pathways, supporting its correlation with aggressive phenotypes (Figure 1D).

Clinical analyses highlighted the prognostic significance of LCN2. In the GSE163209 cohort, the LCN2 signature predicted progression to metastatic disease (AUC = .714; Figure 1E). Kaplan‒Meier survival analyses across three NMIBC cohorts revealed significantly poorer progression‐free survival for patients with high LCN2 expression. In GSE163209, elevated LCN2 levels were also linked to a higher rate of metastatic progression (Figure 1F‐I). Likewise, in The Cancer Genome Atlas (TCGA) and GSE13507 cohorts, elevated LCN2 expression was associated with poor cancer‐specific survival and increased metastasis (Figure 1J‐M). These results underscore LCN2 as a secreted protein linked to BC progression and as a clinically relevant biomarker for disease monitoring and prognosis.

We investigated the functional role of LCN2 by modulating its expression. LCN2 overexpression resulted in increased proliferation, invasion and migration (Figure 2A‐F) and altered epithelial‒mesenchymal transition‐related factors (Figure 2G,H). CM from LCN2‐overexpressing cells and recombinant human LCN2 (rhLCN2) enhanced motility of 5637 cells (Figure 2I‐M). In mice, LCN2 overexpression increased tumour formation, with higher Ki67, LCN2, MMP3 and CD31 expression in tumour tissues (Figure 3A‐C), and promoted lung metastasis supported by CD31 immunoreactivity (Figure 3D‐F).

**FIGURE 2:**
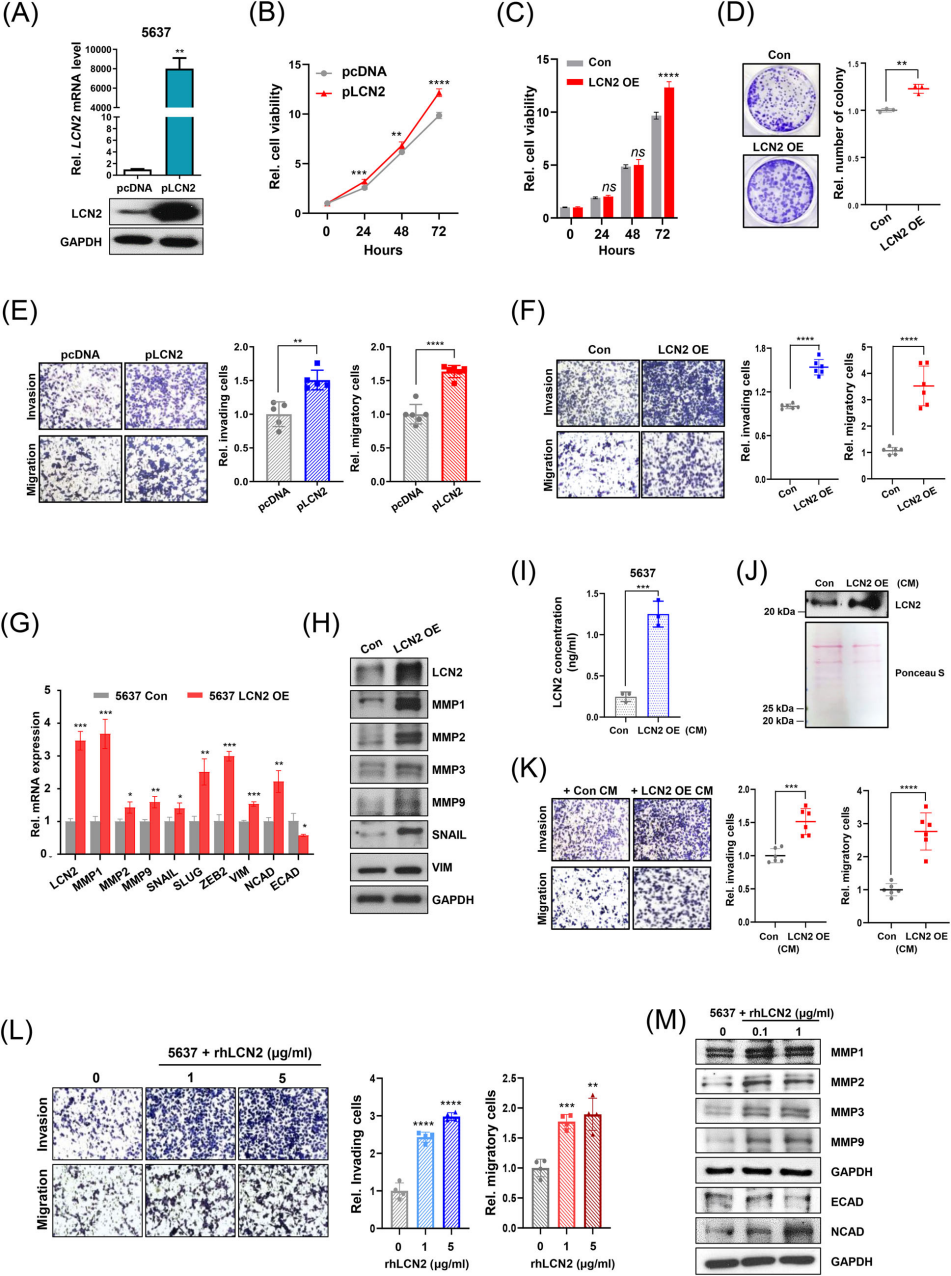
LCN2 regulates cell proliferation and motility in bladder cancer cells. (A) LCN2 mRNA and protein expression levels were analysed in 5637 cells transfected with either the pcDNA or pLCN2 plasmid (*n* = 3). (B) Cell viability was assessed in 5637 cells transfected with pcDNA or pLCN2 plasmid using an MTT (3‐(4,5‐Dimethylthiazol‐2‐yl)‐2,5‐diphenyltetrazolium bromide) assay (*n* = 6). (C) MTT assay was performed to evaluate the viability of 5637 control (Con) and LCN2 overexpression (OE) cell lines (*n* = 6). (D) A colony formation assay was conducted to assess the proliferative ability of 5637 Con and LCN2 OE cells (*n* = 3). (E) The invasion (*n* = 5) and migration (*n* = 6) of transiently LCN2‐overexpressing cells were evaluated using a Boyden chamber assay. (F) A Boyden chamber assay was conducted to examine the invasive and migratory abilities of 5637 Con and LCN2 OE cells (*n* = 6). (G and H) The expression of cell motility‐related factors in 5637 Con and LCN2 OE cells was analysed using (G) qRT‐PCR and (H) Western blot analysis (*n* = 3). (I) LCN2 concentration in conditioned medium (CM) from 5637 Con and LCN2 OE cells was measured using ELISA (*n* = 3). (J) Secreted LCN2 levels in CM from 5637 Con and LCN2 OE cells were confirmed by Western blot analysis, with the Ponceau S‐stained membrane indicating that equal amounts of protein were loaded. (K) The effects of CM derived from 5637 Con and LCN2 OE cells on cell invasion and migration were assessed using a Boyden chamber assay (*n* = 6). (L) The effect of recombinant human LCN2 protein (rhLCN2) on cell invasion and migration was examined using a Boyden chamber assay (*n* = 4). (M) Western blot analysis was performed to investigate the regulation of cell motility‐related factors in 5637 cells treated with rhLCN2. Statistical significance was determined using an unpaired two‐tailed Student's *t*‐test (^*^ *p* < .05, ^**^ *p* < .01, ^***^ *p* < .001, ^****^ *p* < .0001, ns: not significant). All experiments were performed in triplicate.

**FIGURE 3:**
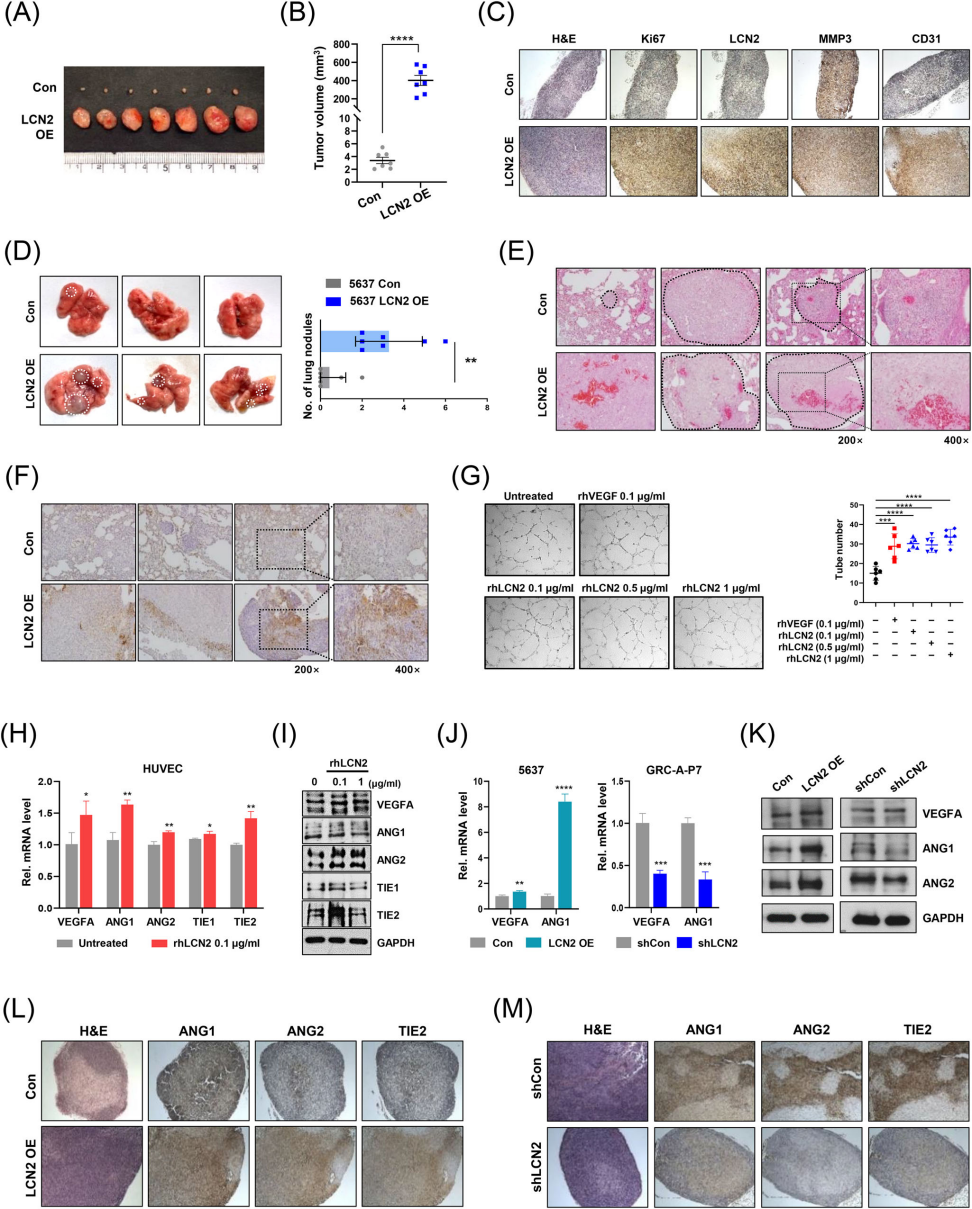
LCN2 promotes tumour formation, metastasis and angiogenesis in vitro and in vivo. (A‒C) A total of 5637 control (Con) and LCN2 overexpression (OE) cells were subcutaneously injected into nude mice (*n* = 7). Representative tumour images are shown in (A), tumour volume measurements are shown in (B) and immunohistochemistry (IHC) for haematoxylin and eosin (H&E), Ki67, LCN2, MMP3 and CD31 in tumour tissues is displayed in (C). (D‒F) A lung metastasis model was employed (*n* = 7). Representative images of lung metastases in mice injected with 5637 Con and LCN2 OE cell lines are shown in (D), H&E‐stained lung sections with nodules indicated by dotted lines are presented in (E) and IHC for CD31 expression in lung tissues is depicted in (F). Images in (E) and (F) were taken at 200× and 400× magnification. (G‒I) Tube formation assays were conducted in HUVECs treated with the indicated concentrations of recombinant VEGF and LCN2 protein (G, *n* = 6), followed by qPCR analysis of VEGFA, ANG1, ANG2, TIE1 and TIE2 (H), and Western blotting of angiogenesis‐related proteins (I). (J and K) LCN2 expression is associated with changes in angiogenic factor levels in BC cell lines. mRNA (J) and protein (K) levels of VEGFA, ANG1 and ANG2 were assessed in 5637 Con, LCN2 OE, 5637GRC‐A‐P7 shCon and shLCN2 cells. (L and M) IHC of angiogenesis‐related markers (H&E, ANG1, ANG2 and TIE2) in tumour tissues from mice injected with 5637 Con or LCN2 OE cells (L) and from mice injected with 5637GRC‐A‐P7 shCon or shLCN2 cells (M). Statistical significance was determined using an unpaired two‐tailed Student's *t*‐test (^*^ *p* < .05, ^**^ *p* < .01, ^***^ *p* < .001, ^****^ *p* < .0001, ns: not significant).

Conversely, LCN2 knockdown in 5637GRC‐A‐P7 cells suppressed proliferation, invasion, migration, tumour growth and metastasis (Figure S2A‒O). Additionally, RNA sequencing data from previously established T24GRC cells also indicated upregulated LCN2 expression (Figure S3A‒C).^9^ Manipulating LCN2 expression in T24GRC, T24 and UC10 cells consistently confirmed that motility depends on LCN2 levels (Figure S3D‒K). These findings collectively demonstrate that LCN2 expression is correlated with BC proliferation, motility and metastatic potential.

Given the role of angiogenesis in metastasis,^10^ we investigated its regulation by LCN2. Treatment with rhLCN2 significantly enhanced tube formation in Human Umbilical Vein Endothelial Cells (HUVECs) (Figure 3G) and upregulated VEGFA, ANG1, ANG2, TIE1 and TIE2 expression (Figure 3H,I). In BC cell lines, LCN2 also influenced the VEGFA, ANG1 and ANG2 expression (Figure 3J,K). Furthermore, the levels of angiogenesis‐related factors in tumour tissues varied based on LCN2 expression (Figure 3L,M). These results suggest that LCN2 is associated with enhanced angiogenic signalling, potentially through the upregulation of VEGFA and ANG, ligands for VEGFR and TIE2 in endothelial cells, and modulation of ANG‒TIE2 signalling.

To assess LCN2 as a liquid biopsy marker for BC diagnosis, we measured LCN2 levels in both serum and urine and evaluated diagnostic performance using receiver operating characteristic (ROC) curve analysis. Serum LCN2 levels were significantly elevated in BC patients compared to controls (AUC = .644; Figure S4A). Notably, these differences were apparent even in low‐stage disease (Tis, Ta and T1), which is often difficult to detect through liquid biopsy. In patients with Tis‐stage, diagnostic performance was particularly robust (AUC = .902; Figure S4B). Additionally, serum LCN2 effectively differentiated between NMIBC and low‐ and high‐grade BC compared to controls. Despite limited sample size, significance was also confirmed for MIBC (Figure S4C,D).

Urinary LCN2 levels were analysed in samples from three independent hospitals. Consistently, urinary LCN2 was elevated in BC patients compared to controls (Figures 4A–D and S5A‒C). A pooled analysis of the three cohorts demonstrated strong diagnostic performance (AUC = .789; Figure 4B). At a cutoff value of 2.974 ng/mL, urinary LCN2 achieved 84.6% sensitivity and 61.5% specificity. Notably, urinary LCN2 effectively distinguished patients of all T stages from controls, showing particularly strong performance in Tis and Ta, and levels tended to increase with advancing stage (Figure 4E). Additionally, urinary LCN2 differentiated NMIBC and MIBC, as well as low‐ and high‐grade tumours from controls (Figure 4F,G). However, its ability to differentiate between NMIBC and MIBC was limited. A significant finding was that postoperative urinary LCN2 levels measured 1 month after surgery were markedly reduced compared to preoperative levels, suggesting a tumour‐derived origin (Figure 4H,I). This indicates that urinary LCN2 has potential as a prognostic biomarker following surgical treatment.

**FIGURE 4:**
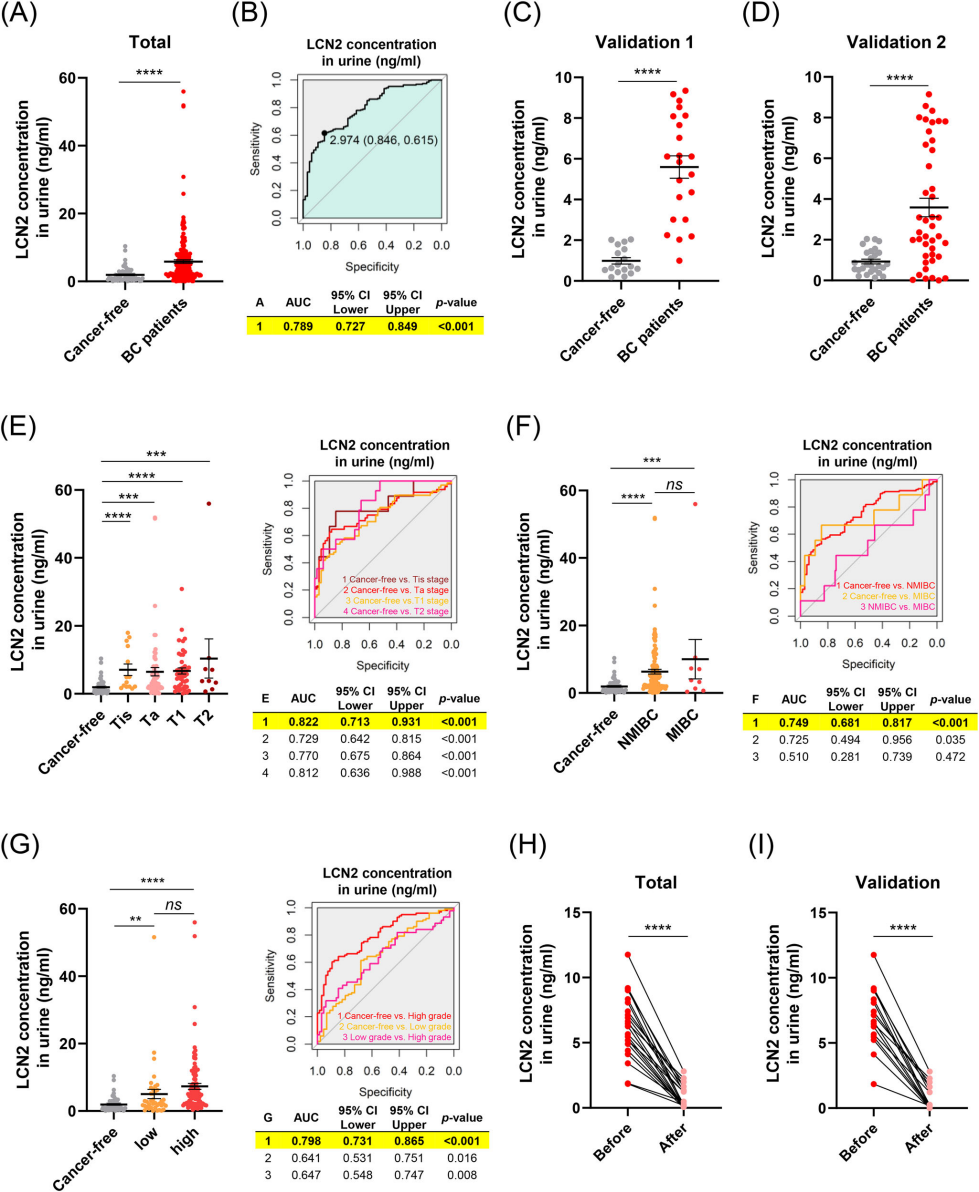
LCN2 as a potential urinary biomarker for the diagnosis of bladder cancer. (A) Overall comparison of urinary LCN2 concentrations between cancer‐free controls (*n* = 65) and bladder cancer (BC) patients (*n* = 204) using pooled ELISA data from discovery and validation cohorts. (B) Receiver operating characteristic (ROC) curve analysis based on the pooled ELISA data shown in panel (A). (C and D) Validation cohorts: urinary LCN2 levels in controls and BC patients from (C) validation 1 (controls, *n* = 17; BC patients, *n* = 22) and (D) validation 2 (controls, *n* = 26; BC patients, *n* = 42). (E) Urinary LCN2 levels according to T stage: controls (*n* = 65), Tis (*n* = 14), Ta (*n* = 62), T1 (*n* = 45) and T2 (*n* = 9). (F) Comparison of urinary LCN2 levels between controls (*n* = 65) and patients with non‐muscle‐invasive bladder cancer (NMIBC) (*n* = 131) or muscle‐invasive bladder cancer (MIBC) (*n* = 9). (G) Comparison of urinary LCN2 levels between controls and patients with low‐grade (*n* = 39) or high‐grade BC (*n* = 98). Panels (E–G) present ELISA results (left) alongside corresponding ROC curves (right). (H) Paired analysis of pre‐ and postoperative urinary LCN2 levels were conducted using pooled data from discovery and both validation cohorts (*n* = 22). (I) Paired analysis of pre‐ and postoperative urinary LCN2 levels were conducted using pooled data from the validation 1 and 2 cohorts (*n* = 16). Statistical significance for ELISA data (panels A–G) was determined using unpaired two‐tailed Student's *t*‐tests, while paired comparisons (panels H–I) were analysed using paired two‐tailed Student's *t*‐tests. ROC curve comparisons were evaluated using the DeLong test for differences in AUC. *p* < .05 was considered statistically significant (^*^ *p* < .05, ^**^ *p* < .01, ^***^ *p* < .001, ^****^ *p* < .0001, ns: not significant).

In conclusion, LCN2 is a promising liquid biopsy biomarker for BC. Elevated levels of LCN2 in both serum and urine, particularly in early‐stage disease, effectively differentiate BC patients from controls. The postoperative decrease in urinary LCN2 levels further indicates its tumour origin and prognostic value. Additionally, LCN2's association with BC motility and angiogenesis positions it as both a biomarker and a potential therapeutic target. Although its effectiveness as a standalone marker is limited, combining LCN2 with other biomarkers in a multimodal approach could improve diagnostic accuracy and facilitate early detection. To confirm LCN2's clinical applicability for BC monitoring, further validation in larger and more diverse populations is essential.

## AUTHOR CONTRIBUTIONS

Mi‐So Jeong, Jeong‐Yeon Mun, Tae‐Nam Kim and Sun‐Hee Leem conceived and designed the study. Mi‐So Jeong, Jeong‐Yeon Mun, Gi‐Eun Yang, Sung Ho Yun, Jae‐Jun Kim and Seo‐Yeong Yoon conducted the experiments. Seung‐Woo Baek and Sang‐Yeop Lee performed the statistical analyses. Tae‐Nam Kim, Jong‐Kil Nam and Hyeok Jun Goh collected clinical samples and provided clinical information. Mi‐So Jeong, Jeong‐Yeon Mun, Gi‐Eun Yang and Seung‐Woo Baek drafted the manuscript. Mi‐So Jeong, Jeong‐Yeon Mun, Gi‐Eun Yang, Seung‐Woo Baek, Seung Il Kim, Yung‐Hyun Choi, Tae‐Nam Kim and Sun‐Hee Leem critically revised and edited the manuscript. All the authors read and approved the final version.

## CONFLICT OF INTEREST STATEMENT

All the authors declare that they have no financial or non‐financial conflicts of interest.

## ETHICS STATEMENT

The study involving human participants was approved by the Institutional Review Boards of Pusan National University (IRB no. H‐1706‐002‐007) and Pusan National University Yangsan Hospital (IRB no. 55‐2023‐003). Written informed consent was obtained from all participants before sample collection. Animal experiments were approved by the Institutional Animal Care and Use Committee (IACUC) of Dong‐A University (approval no. DIACUC‐20‐41) and were conducted in accordance with institutional guidelines and national regulations for the care and use of laboratory animals.

## Supporting information



Supporting Information

## Data Availability

The RNA sequencing dataset used in this study is publicly available from the NCBI Gene Expression Omnibus (GEO) under accession number GSE210954. The LC–MS/MS proteomic data generated during this study can be requested from the corresponding author upon reasonable request. Publicly available bladder cancer gene expression datasets (UROMOL2021, GSE32894, GSE163209, GSE13507 and TCGA) were obtained from the GEO and GDC databases as described in the Methods section.
