# Supplementary material for: Lipocalin 2 as a potential liquid biopsy marker for early detection of bladder cancer
Source: Clin Transl Med. 2025 Dec 9;15(12):e70540. doi: 10.1002/ctm2.70540 (PMC12687296; doi:10.1002/ctm2.70540)
Supplement: Supplementary file 1 — Supporting Information [file CTM2-15-e70540-s001.doc]

**Lipocalin 2 as a Potential Liquid Biopsy Marker for Early Detection of Bladder Cancer**

Mi-So Jeong^1,2,12^, Jeong-Yeon Mun^3,12^, Gi-Eun Yang^1,4^, Seung-Woo Baek^5^, Sang-Yeop Lee^6^, Sung Ho Yun^7^, Seung Il Kim^6^, Jae-Jun Kim^1,4^, Seo-Yeong Yoon^1,4^, Jong-Kil Nam^8^, Yung-Hyun Choi^9^, Hyeok Jun Goh^10^, Tae-Nam Kim^11✉^, Sun-Hee Leem^1,4,✉^

^1^Department of Biomedical Sciences, Dong-A University, Busan 49315, Korea. ^2^Research Center, Dongnam Institute of Radiological & Medical Sciences (DIRAMS), Busan, 46033, Korea. ^3^Department of Pathology and Cell Biology, Columbia University Medical Center, New York, NY 10032, USA. ^4^Department of Health Sciences, The Graduated of Dong-A University, Busan 49315, Korea. ^5^Genomic Medicine Research Center, Korea Research Institute of Bioscience and Biotechnology (KRIBB), Daejeon 34141, Korea. ^6^Research Center for Bioconvergence Analysis, Korea Basic Science Institute, Ochang, 28119, Korea. ^7^Center for Research Equipment, Korea Basic Science Institute, Ochang, 28119, Republic of Korea. ^8^Department of Urology, Pusan National University Yangsan Hospital, Pusan National University School of Medicine, Research Institute for Convergence of Biomedical Science and Technology, Yangsan 50612, Korea. ^9^Department of Biochemistry, College of Oriental Medicine, Anti-Aging Research Center, Dong-eui University, Busan 47227, Korea. ^10^Department of Urology, Dong-A University College of Medicine, Busan 49201, Korea. ^11^Department of Urology, Pusan National University Hospital, Pusan National University School of Medicine, Biomedical Research Institute and Pusan National University Hospital, Busan 49241, Korea. ^12^These authors contributed equally to this manuscript : Mi-So Jeong, Jeong-Yeon Mun. ^✉^Corresponding authors: bigman1995@hanmail.net, shleem@dau.ac.kr

**1. Methods and Materials**

**1.1 Cell culture**

Cell lines 5637 and 5637GRC were cultured in RPMI 1640 medium with 10% FBS and 1% penicillin/streptomycin (Capricorn Scientific GmbH, Germany). All cell lines were incubated at 37℃ in a humidified atmosphere of 5% CO_2_.

**1.2 Preparation of cell-cultured conditioned medium (CM)**

The 5637GRC-A cells were seeded, incubated for 24 hours, and treated with serum-free RPMI 1640 medium for 6 hours. The CM was filtered (0.45 μm, Millipore, MA) and concentrated using VIVASPIN column (GE Healthcare, USA).

**1.3 Proteomic analysis by liquid chromatography tandem mass spectrometry (LC−MS/MS)**

Protein concentration in CM was determined by BCA assay (Thermo Fisher Scientific, USA). Concentrated CM samples were loaded on a 12% SDS−PAGE gel. The gel was stained with Coomassie Brilliant Blue R−250 buffer (Thermo Fisher Scientific) and excised into gel pieces covering the entire molecular weight range. In-gel digestion was performed according to a previously described method ^1^. All MS and MS/MS data were obtained using Q−Exactive Plus mass spectrometer in data-dependent mode, and analysed using MASCOT 2.4 software. UniProt human proteome sequences (UP000005640) were used as the database, and a cutoff value of a *p*−value less than 0.05 was applied. Protein quantities were calculated using the exponentially modified protein abundance index, and denoted as mol%. LC−MS/MS analysis was performed at least three times for each sample.

**1.4 Cell transfection and the establishment of stable cell lines**

Cells were transfected with pcDNA6/V5−HisA (pcDNA) and pcDNA6-LCN2 (pLCN2) plasmids, or scrambled siRNA (scRNA) and siLCN2 (Integrated DNA Technologies, USA) using jetPRIME reagent (Polyplus, France). Cells were harvested after 24 h. To establish stable knockdown cell lines, lentiviruses were produced using pLKO.1−puro (shCon) or pLKO.1−LCN2 (shLCN2, Clone ID TRCN0000060291, Sigma−Aldrich, USA), psPAX2, and pMD2.G vectors in 293T cell line. The 5637GRC−A−P7 cell line was infected with lentiviruses at an MOI value, followed by puromycin selection.

**1.5 Cell proliferation assay**

Cells were seeded in 96−well plates for the cell proliferation assay. Cell viability was measured at each time point after treatment with thiazolyl blue tetrazolium bromide (MTT, Sigma−Aldrich, USA). Absorbance was measured at 540 nm using spectrophotometer microplate reader (Victor 3). For clonogenic assay, cells were seeded in 12−well plates. After 7 days, cells were stained with 0.5% crystal violet and counted using ImageJ program (National Institutes of Health; NIH, USA).

**1.6 Cell motility assay**

The ability of cell invasion and migration was determined using Boyden chamber assay. Membranes were coated with Matrigel (BD biosciences, USA) for invasion assay, or type I collagen (Corning, USA) for migration assay. Cells were seeded in serum-free medium in the upper chamber, while the bottom chamber contained medium with 1% FBS for invasion, or 10% FBS for migration. After 24 hours, cells were fixed and stained using Diff−Quik staining solution (Sysmex, Japan). Cell counts were performed using ImageJ.

**1.7 RNA extraction and quantitative real-time PCR (qRT−PCR)**

Total RNA was isolated using RNAiso Plus (Takara, Japan). cDNA was reverse transcribed from the total RNA using PrimeScript^TM^ RT Master Mix (Takara). qRT−PCR was performed with TB Green^TM^ Premix Ex Taq (Tli RNaseH Plus, Takara) on a CFX96 real time PCR detection system (Bio−Rad, USA). Table S1 provides the detailed primer sequences.

**1.8 Western blot analysis**

Protein concentration was measured using the BCA assay kit (Thermo Fisher Scientific, USA). Total proteins were separated by SDS−PAGE, and transferred to nitrocellulose membrane (GE healthcare, USA). The membrane was incubated with the primary antibodies listed in Table S2. The membrane was incubated with horseradish peroxidase (HRP)-conjugated secondary antibody. Immunoreactivity was detected using Western Bright^TM^ ECL reagent (Advansta, USA).

**1.9 Patient information**

The study protocol was approved by the Institutional Review Boards of Pusan National University (IRB No. H−1706−002−007), Pusan National University Yangsan Hospital (IRB No. 55−2023−003). All procedures were conducted in accordance with the Declaration of Helsinki. Serum and urine samples were collected from BC patients and cancer-free controls across three independent cohorts: a discovery cohort, and two validation cohorts. Serum samples were obtained exclusively from the discovery cohort, whereas urine samples were collected from all three cohorts. Cancer-free controls included healthy individuals and patients with non-malignant conditions, such as lower urinary tract symptoms, urolithiasis, hematuria, urinary tract infections, hypertension, and diabetes. The discovery cohort comprised 154 BC patients and 65 cancer-free controls. Validation cohort 1 included 22 BC patients and 17 controls, while validation cohort 2 included 42 BC patients and 26 controls (Table S3).

**1.10 Enzyme-linked immunosorbent assay (ELISA)**

Blood samples were collected in vacutainers and allowed to clot at room temperature, followed by centrifugation at 3,000 rpm for 10 min at 4°C. Urine samples were collected and centrifuged to remove debris. LCN2 concentrations were measured using the Human Lipocalin−2/NGAL DuoSet ELISA kit (R&D systems, USA). LCN2 concentrations were calculated by subtracting the absorbance at 540 nm from that at 450 nm, and applying the resulting value to the standard curve.

**1.11 Recombinant human LCN2 (rhLCN2) protein**

Cell line 5637 was suspended in serum-free RPMI 1640 medium containing rhLCN2 (R&D systems, USA), and their invasion and migration abilities examined by Boyden chamber assay. The 5637 cell line and human umbilical vein endothelial cells (HUVECs) were serum-starved for 6 hours, and treated with rhLCN2 at concentrations of 0.1 and 1 μg/ml. After 24 hours, RNA and proteins levels were examined.

**1.12 Mouse xenograft**

The animal study protocol was approved by the Institutional Animal Care and Use Committee (IACUC) of Dong−A University (Approval No. DIACUC−20−41). All animal experiments were conducted in accordance with the institutional guidelines and relevant regulations for the care and use of laboratory animals. For tumor xenograft transplantation, cell mixture with Matrigel was subcutaneously injected into the flank of 6-week-old male BALB/c nude mice (Orient bio, Korea). After 8 weeks, tumor tissues were harvested. Tumor volume was calculated using following formula: tumor volume (mm^3^) = (length [mm] × width^2^ [mm^2^])/2. For tumor metastasis experiment, cells were injected into the tail vein of 6-week-old male BALB/c nude mice. After 8 weeks, mouse lung tissues and metastasized tumor tissues were collected. Tumor burdens were confirmed by H&E staining of paraffin-embedded tissue sections.

**1.13 Immunohistochemistry (IHC)**

The mouse tumor tissue and lung tissue were fixed in formalin solution (Sigma−Aldrich, USA) and embedded in paraffin. IHC was performed as previously described ^2^. Primary antibodies used in IHC were listed in Table S2. The staining intensity of the tissue was examined by the optical microscopy.

**1.14 Tube formation assay**

HUVECs were cultured in EBM−2 (endothelial cell basal medium-2) supplemented with EGM−2 MV SingleQuots (Lonza, USA). For tube formation assays, matrigel was mixed with serum-free EBM−2 medium and coated in 96-well plate. After 1 hour, HUVECs treated with rhVEGF or rhLCN2 was seeded. Tube formation was examined and imaged using an optical microscopy.

**1.15 Public datasets of NMIBC and MIBC patients**

Clinical and gene expression data for NMIBC and MIBC patients were obtained from public databases: UROMOL2021 (European Genome-Phenome Archive, EGAS0001004693) ^3^, bladder cancer microarray studies (Gene Expression Omnibus (GEO): GSE32894, GSE163209, and GSE13507) ^4-6^, and The Cancer Genome Atlas (TCGA), via Xena Browser platform (https://xenabrowser.net)). The cohorts used in Figure 1, specifically UROMOL2021, GSE163209, GSE32894, TCGA, and GSE13507, are all independent, publicly available datasets and do not have overlapping patient samples.

**1.16 Statistical analysis**

Data are presented as the mean ± SD for cell-based experiments and as the mean ± SEM for clinical and ELISA data. The exact number of biological and/or technical replicates (*n* value) for each experiment is indicated in the corresponding figure and table legends. Statistical significance was determined using unpaired or paired two-tailed Student’s t−tests, as appropriate, in GraphPad Prism 9. Receiver operating characteristic (ROC) curve comparisons were evaluated using the DeLong test for area under the curve (AUC) differences. *P* < 0.05 was considered statistically significant (**p* < 0.05, ***p* < 0.01, ****p* < 0.001, *****p* < 0.0001; ns = not significant). KEGG pathway analysis was conducted with DAVID (false discovery rate [FDR] < 0.25), while PROGENy was used to identify pathway response genes ^7^. Kaplan–Meier plots were evaluated using the log−rank test, and clinical data comparisons were assessed using the chi−square or Fisher’s exact tests. All statistical analyses were performed in R 4.2.1.

**References**

1. Shevchenko A, Tomas H, Havlis J, Olsen JV, Mann M. In-gel digestion for mass spectrometric characterization of proteins and proteomes. *Nat Protoc*. 2006;1(6):2856-60. doi:10.1038/nprot.2006.468

2. Mun JY, Baek SW, Jeong MS, et al. Stepwise molecular mechanisms responsible for chemoresistance in bladder cancer cells. *Cell Death Discov*. Nov 7 2022;8(1):450. doi:10.1038/s41420-022-01242-8

3. Lindskrog SV, Prip F, Lamy P, et al. An integrated multi-omics analysis identifies prognostic molecular subtypes of non-muscle-invasive bladder cancer. *Nat Commun*. Apr 16 2021;12(1):2301. doi:10.1038/s41467-021-22465-w

4. Lee JS, Leem SH, Lee SY, et al. Expression signature of E2F1 and its associated genes predict superficial to invasive progression of bladder tumors. *J Clin Oncol*. Jun 1 2010;28(16):2660-7. doi:10.1200/JCO.2009.25.0977

5. Hurst CD, Cheng G, Platt FM, et al. Stage-stratified molecular profiling of non-muscle-invasive bladder cancer enhances biological, clinical, and therapeutic insight. *Cell Rep Med*. Dec 21 2021;2(12):100472. doi:10.1016/j.xcrm.2021.100472

6. Sjodahl G, Lauss M, Lovgren K, et al. A molecular taxonomy for urothelial carcinoma. *Clin Cancer Res*. Jun 15 2012;18(12):3377-86. doi:10.1158/1078-0432.CCR-12-0077-T

7. Schubert M, Klinger B, Klunemann M, et al. Perturbation-response genes reveal signaling footprints in cancer gene expression. *Nat Commun*. Jan 2 2018;9(1):20. doi:10.1038/s41467-017-02391-6


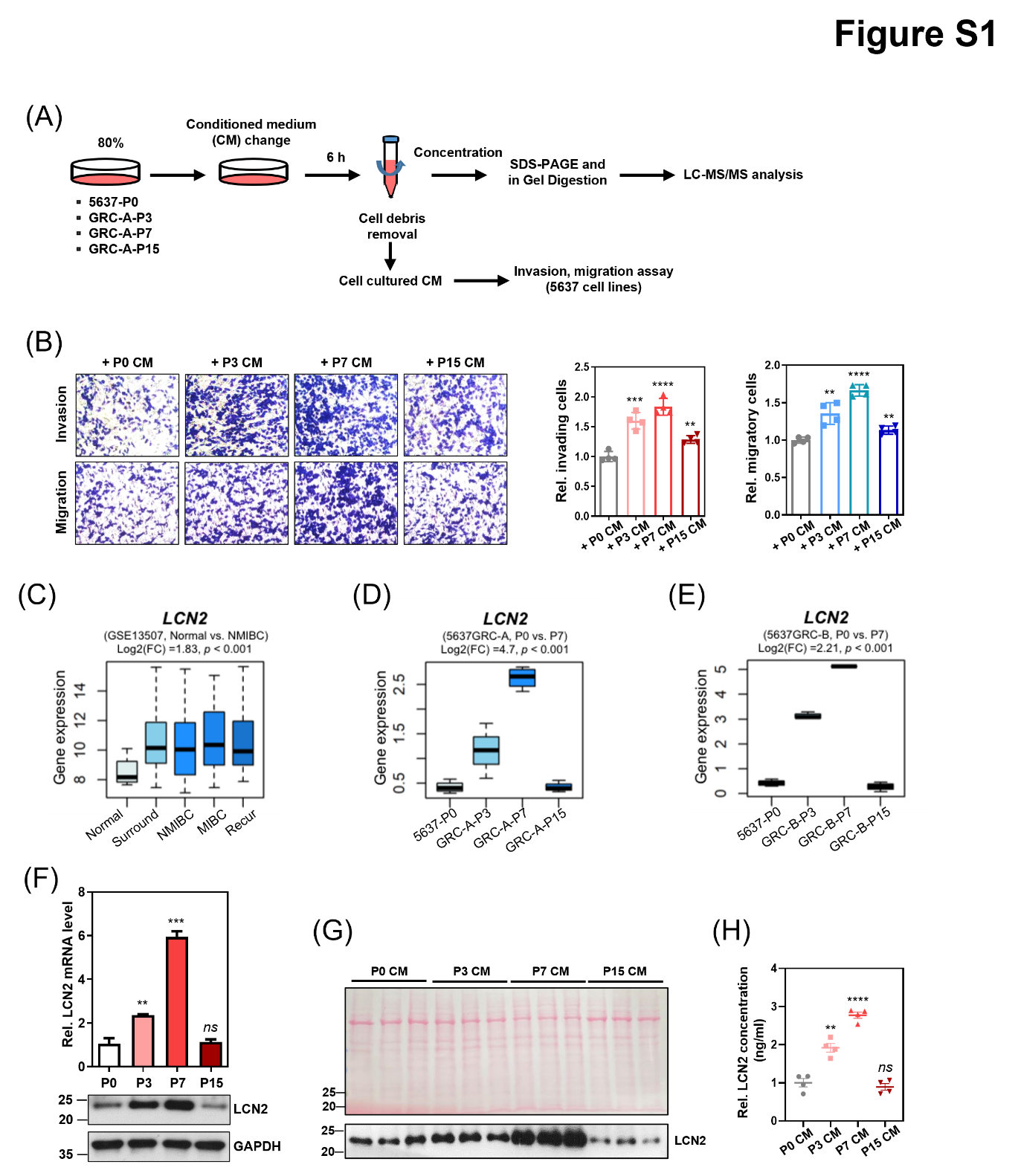


**Figure S1. LCN2 is identified as a metastatic biomarker that promotes motility in 5637GRC−A cell lines.**

(A) Schematic illustrating the preparation of conditioned medium (CM) derived from 5637GRC−A cell lines. (B) The upper chamber was filled with 5637 cell line suspended in a 1:1 mixture of CM and fresh serum-free medium. The lower chamber contained RPMI 1640 medium supplemented with either 1% FBS (for invasion) or 10% FBS (for migration). After 24 hours of incubation, cells were stained to assess motility (n = 4). (C) LCN2 expression levels in the bladder cancer cohort GSE13507. (D) LCN2 expression levels in 5637GRC−A cell lines. (E) LCN2 expression levels in 5637GRC−B cell lines. (F) LCN2 mRNA and protein levels in 5637GRC−A cell lines were examined using qRT−PCR and western blot analysis, respectively, with GAPDH as a control (n = 3). (G) Total protein concentration in CM derived from 5637GRC−A cell lines was quantified using a BCA assay. Secreted LCN2 levels were analyzed by western blot, with Ponceau S staining confirming equal loading of samples (n = 3). (H) The concentration of secreted LCN2 in CM was quantified by ELISA (n = 4). CM samples were collected and concentrated. Absorbance was measured at 450 nm and 540 nm using a Victor 3 plate reader. (*, *p* < 0.05; **, *p* < 0.01; ***, *p* < 0.001; ****, *p* < 0.0001; ns, not significant; Rel., relative)


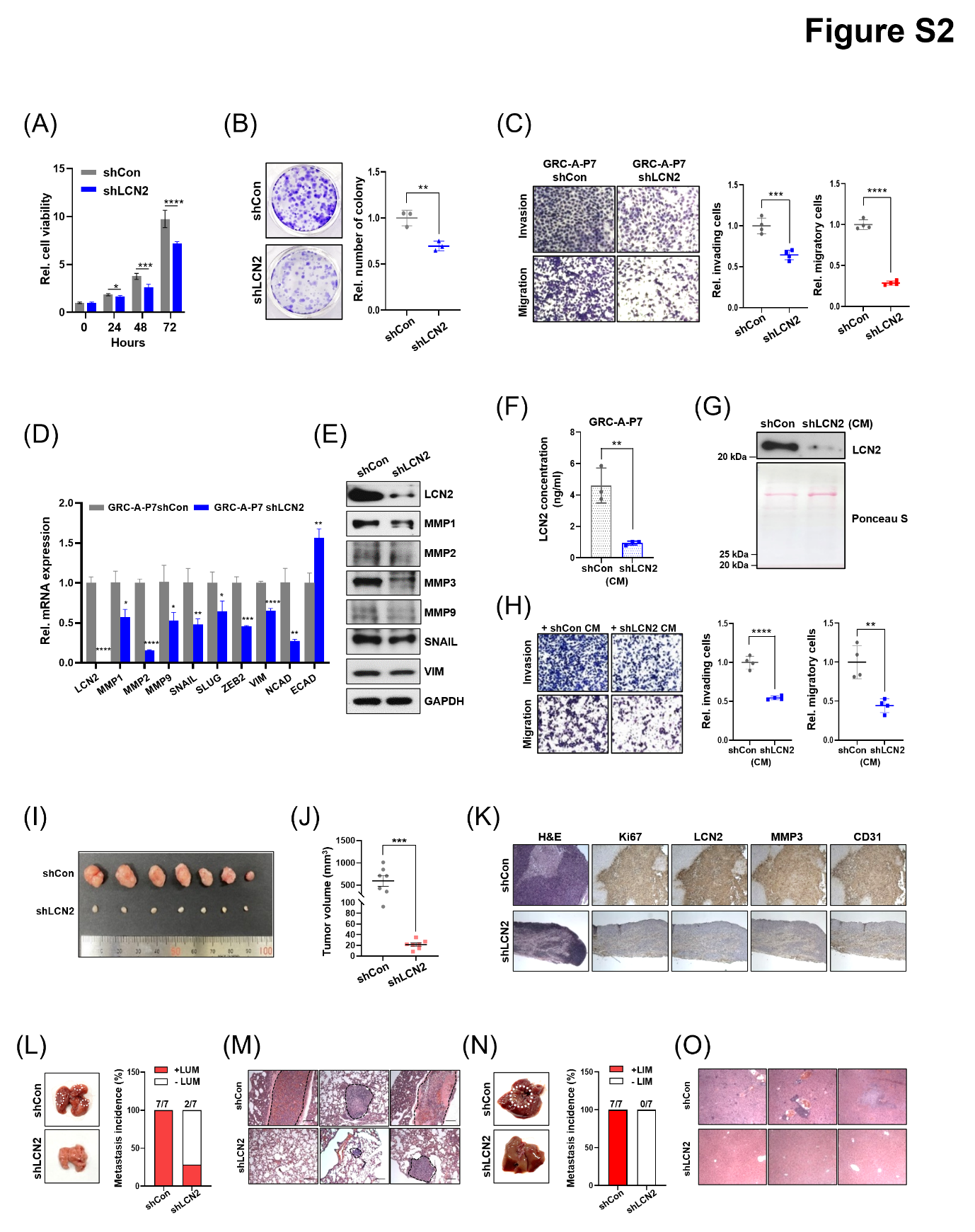


**Figure S2. LCN2 knockdown suppresses bladder cell growth, motility, and metastasis.**

(A) Cell viability of 5637GRC−A−P7 cells stably transduced with LCN2 shRNA (shLCN2) or control shRNA (shCon) was evaluated by MTT assay (n = 6). (B) Colony formation ability of 5637GRC−A−P7 shCon and shLCN2 cells (n = 3). (C) Migration and invasion capacities were evaluated using a Boyden chamber assay (n = 4). (D-E) Expression levels of motility-associated genes in shCon and shLCN2 cells were analyzed by (D) qRT−PCR (n = 3) and (E) western blotting. (F-G) Secreted LCN2 protein levels in conditioned medium (CM) from shCon and shLCN2 cells were quantified by (F) ELISA (n = 3) and (G) western blot. (H) Migratory and invasive abilities of parental 5637 cells treated with CM from 5637GRC-A-P7 shCon and shLCN2 cells (n = 4). (I) Representative images of tumor tissues formed by shCon and shLCN2 cells in xenograft models. (J) Tumor volumes were measured with calipers (n = 7). (K) Immunohistochemical (IHC) staining of tumor tissues for the indicated markers. Images were captured at 100× magnification. (L) Representative images of lung metastases in mice injected with shCon or shLCN2 cells; tumor nodules are outlined with white dotted lines. The incidence (%) of lung metastases is shown. (M) Hematoxylin and eosin (H&E) staining of lung tissues from mice injected with shCon or shLCN2 cells. (N) Representative images of liver metastases; white dotted lines indicate tumor nodules. The incidence (%) of liver metastases is shown (n = 7). (O) H&E staining of liver tissues from mice injected with shCon or shLCN2 cells. Abbreviations: LUM, lung metastasis; LIM, liver metastasis.


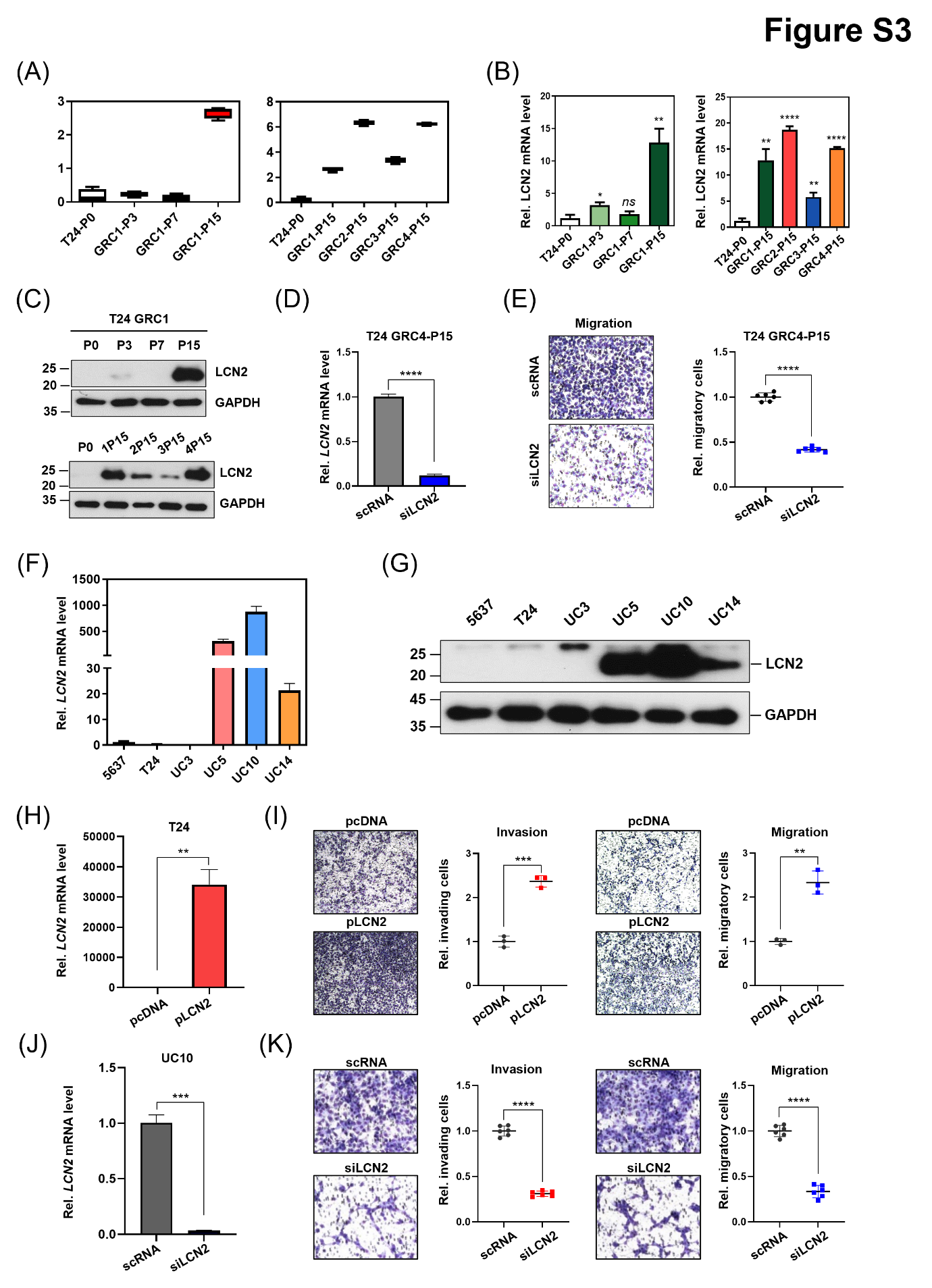


**Figure S3. LCN2 regulates cell motility in BC cell lines.**

(A) LCN2 expression levels in RNA sequencing data from T24GRC cell lines. (B, C) LCN2 mRNA and protein levels were analyzed by (B) qRT−PCR (n = 3) and (C) western blotting, respectively, in T24GRC cell lines (n = 3). (D) LCN2 mRNA expression was measured by qRT-PCR in T24GRC4−P15 cells following LCN2 siRNA transfection (n = 3). (E) The migratory capacity of T24GRC4−P15 cells transfected with either scRNA or siLCN2 was evaluated (n = 6). (F, G) LCN2 (F) mRNA and (G) protein levels were assessed in human BC cell lines. (H) LCN2 mRNA expression was analyzed after LCN2 overexpression in T24 cells (n = 3). (I) The invasive and migratory abilities of T24 cells transfected with either pcDNA or pLCN2 were assessed using a Boyden chamber assay (n = 3). (J) LCN2 mRNA expression was measured after knockdown in UC10 cells (n = 3). (K) The invasive and migratory capacities of UC10 cells transfected with either scRNA or siLCN2 were evaluated using a Boyden chamber assay (n = 6).


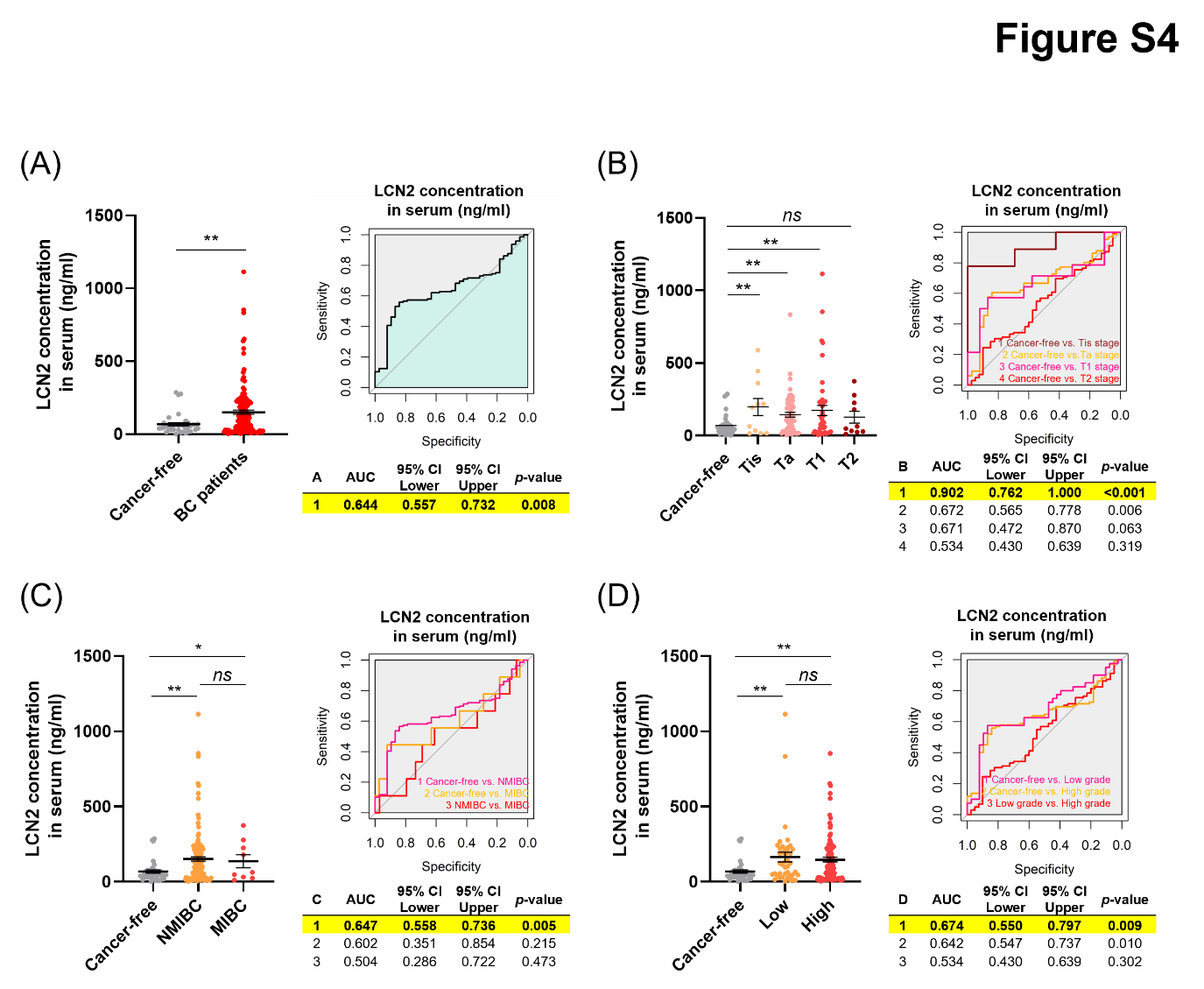


**Figure S4. Diagnostic performance of LCN2 concentration in serum.**

(A) Serum concentrations of LCN2 were compared to cancer-free controls (n = 38) with bladder cancer (BC) patients (n = 145). (B) Serum LCN2 levels were analyzed according to bladder cancer T−stage. Controls (n = 38), Tis (n = 11), Ta (n = 61), T1 (n = 46), T2 (n = 10). (C) Serum LCN2 levels were measured in cancer-free controls (n = 38) and patients with NMIBC (n = 136) or MIBC (n = 9). (D) Serum LCN2 concentrations were compared between cancer-free controls (n = 38) and BC patients stratified by tumor grade. Low-grade (n = 42), high-grade (n = 102). For all analyses, ELISA results are shown in the left panel, while corresponding ROC curves are presented in the right panel.


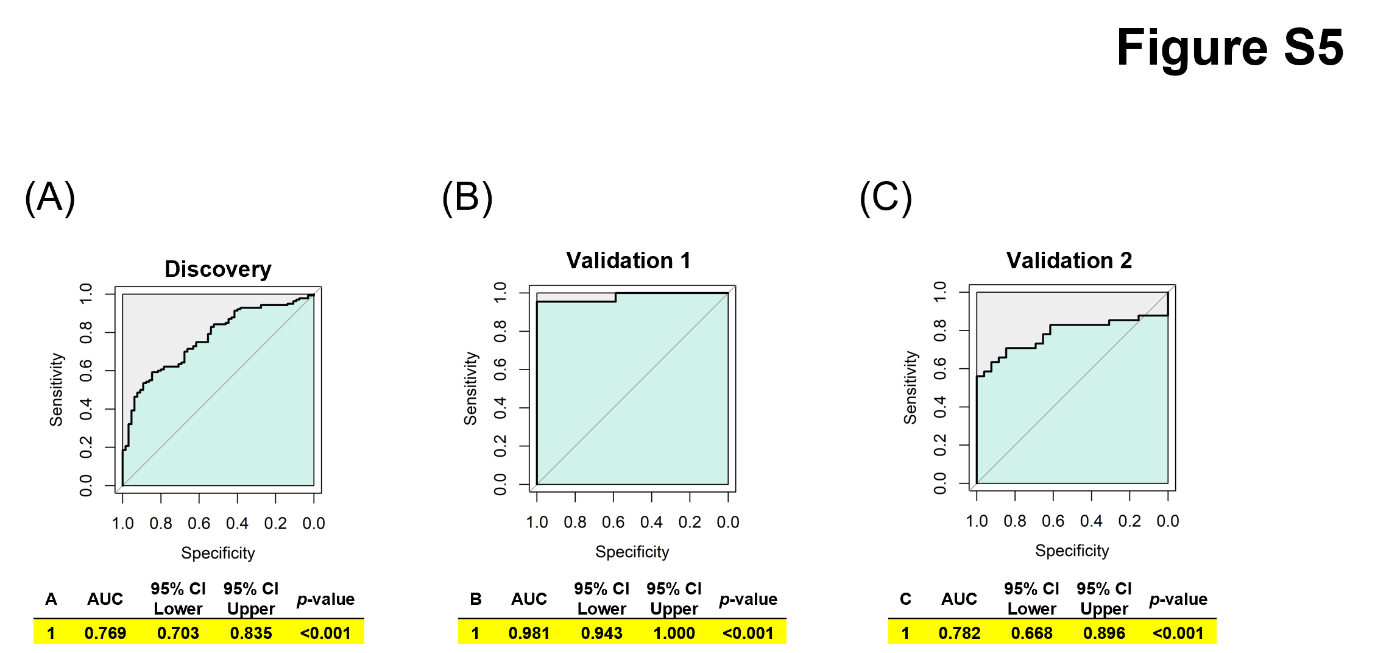


**Figure S5. Diagnostic performance of urinary LCN2 concentrations across three independent cohorts.**

(A−C) show ROC curve analyses evaluating the ability of urinary LCN2 levels to distinguish BC patients from cancer-free controls in (A) discovery, (B) validation 1, and (C) validation 2 cohorts.


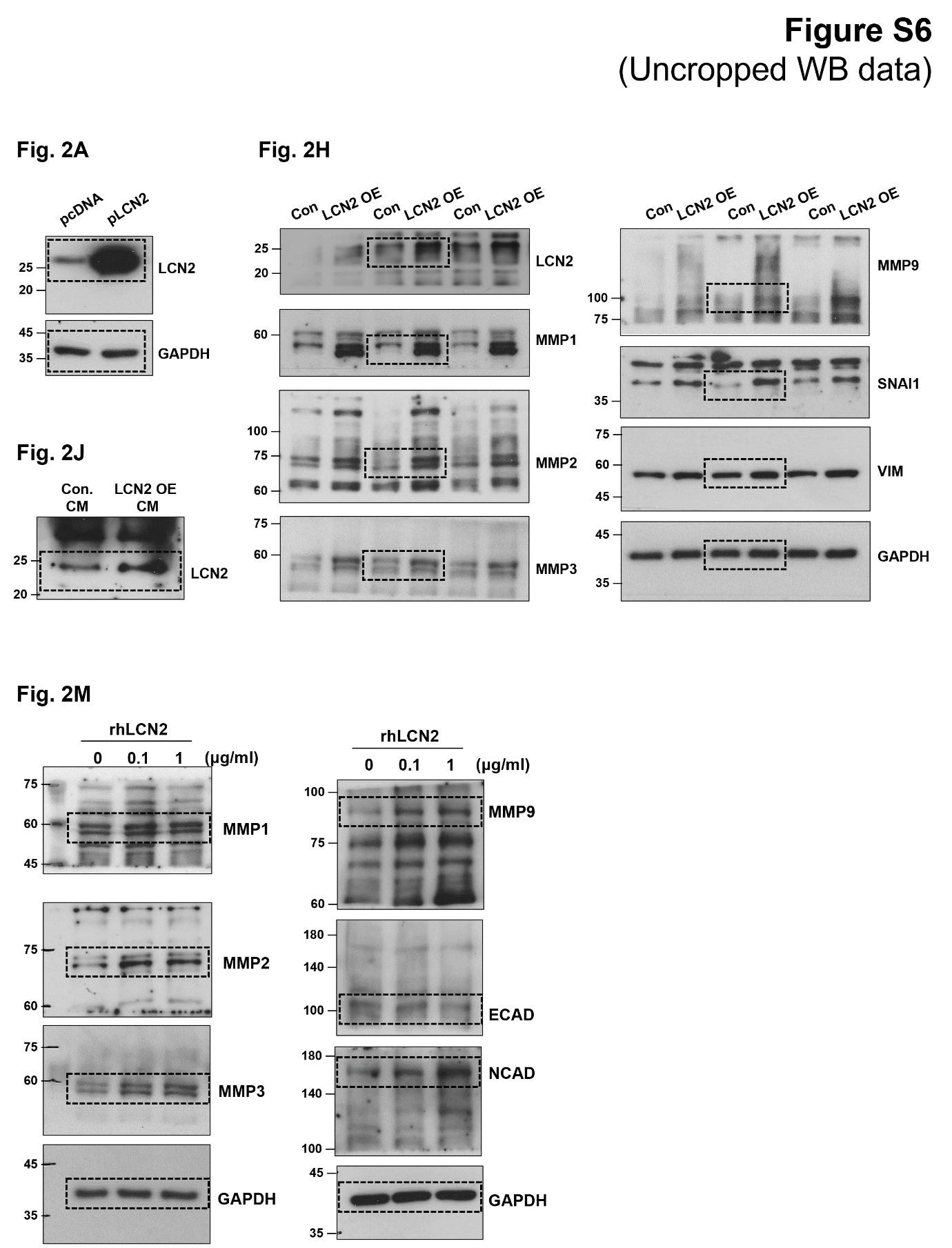


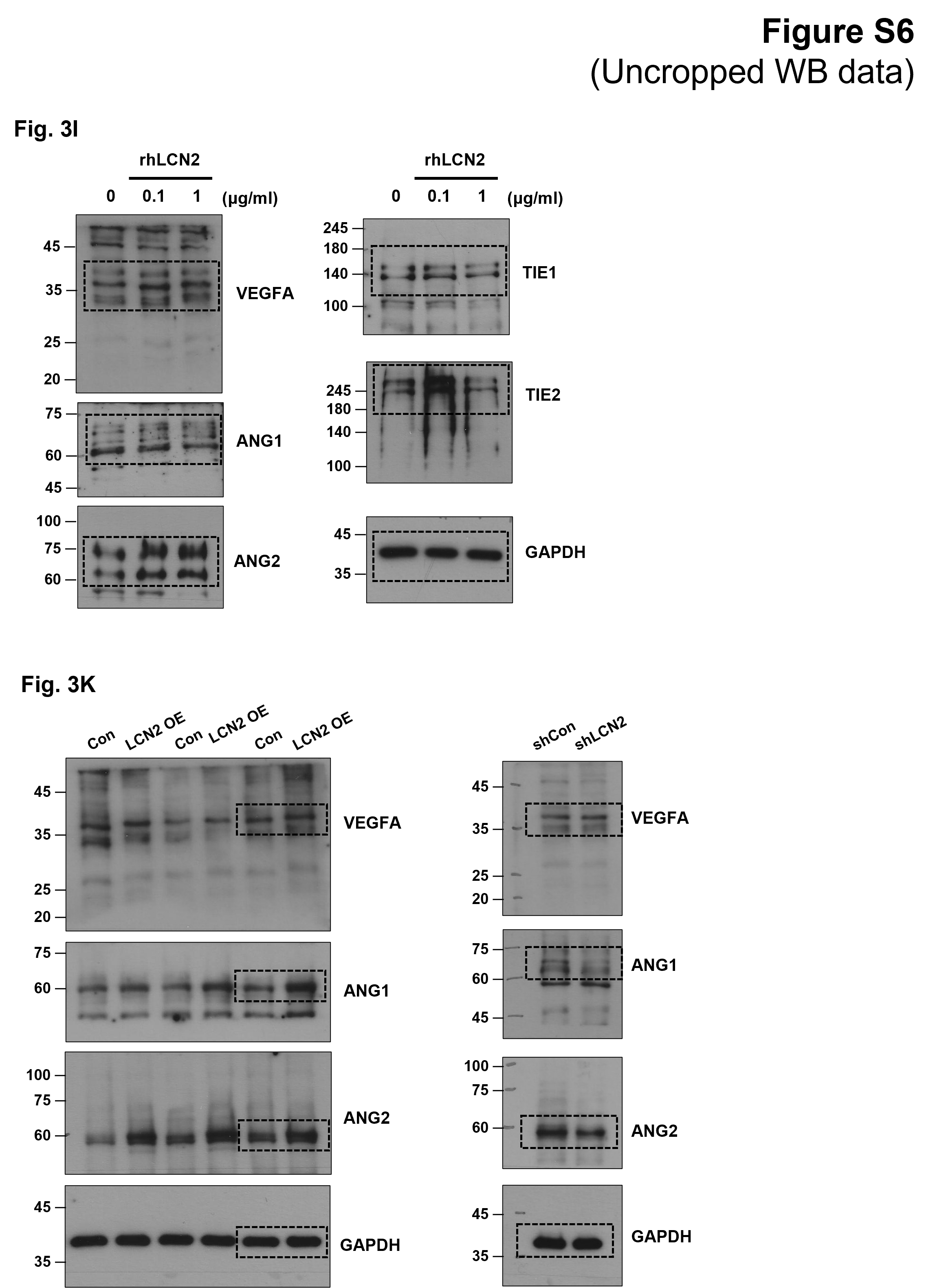


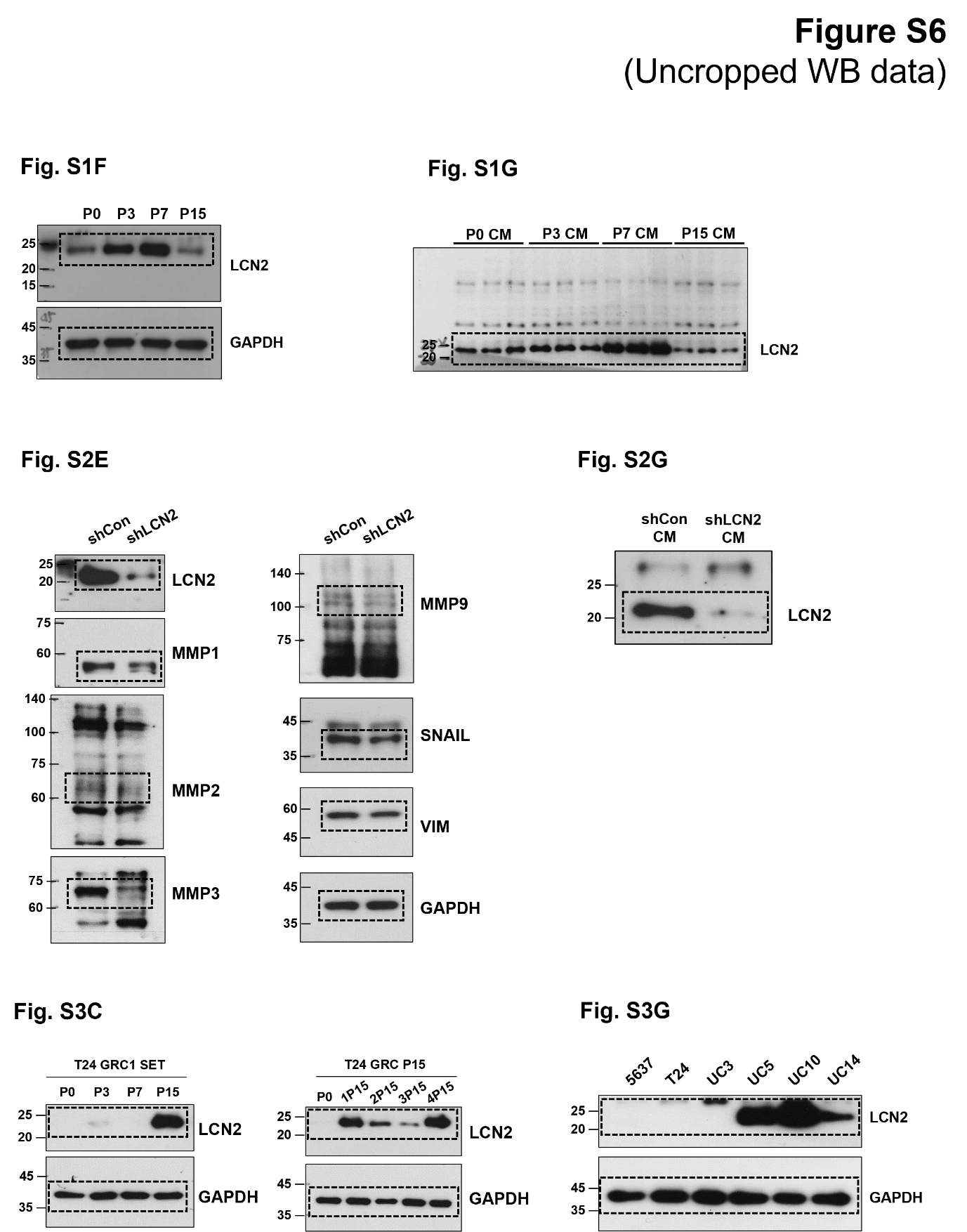


**Table S1.** Sequences of qPCR primer pairs

| Gene | Sequence (5'-3') | Product size |
| --- | --- | --- |
| *LCN2* | F: 5'-AGACAAAGACCCGCAAAAG-3' | 83 bp |
|  | R: 5'-TAAACAGGACGGAGGTGAC-3' |  |
| *MMP1* | F: 5'-TTTGGCTTCCCTAGAACTGTG-3' | 146 bp |
|  | R: 5'-GCTATCATTTTGGGATAACCTGG-3' |  |
| *MMP2* | F: 5'-GCGGCGGTCACAGCTACTT-3' | 71 bp |
|  | R: 5'-CACGCTCTTCAGACTTTGGTTCT-3' |  |
| *MMP9* | F: 5'-CCTGGAGACCTGAGAACCAATC-3' | 79 bp |
|  | R: 5'-CCACCCGAGTGTAACCATAGC-3' |  |
| *SNAIL* | F: 5'-CCACAAGCACCAAGAGTC-3' | 136 bp |
|  | R: 5'-TGGCAGTGAGAAGGATGT-3' |  |
| *SLUG* | F: 5'-TTCACTCCGAAGCCAAATG-3' | 121 bp |
|  | R: 5'-TCTCTCTGTGGGTGTGTG-3' |  |
| *ZEB2* | F: 5'-ATCGTGTAACAAAGATGAAGAAA-3' | 142 bp |
|  | R: 5'-TCACAAATGTCTCAAGTTCTAAA-3' |  |
| *VIM* | F: 5'-AGGCAAAGCAGGAGTCCACTGA-3' | 100 bp |
|  | R: 5'-ATCTGGCGTTCCAGGGACTCAT-3' |  |
| *NCAD* | F: 5'-CCTGCTTATCCTTGTGCTG-3' | 144 bp |
|  | R: 5'-TCTTCTTCTCCTCCACCTTC-3' |  |
| *ECAD* | F: 5'-TTCCTCCCAATACATCTCCC-3' | 142 bp |
|  | R: 5'-TTGATTTTGTAGTCACCCACC-3' |  |
| *VEGFA* | F: 5'-AAAACGAAAGCGCAAGAAATCC-3' | 209 bp |
|  | R: 5'-TTAACTCAAGCTGCCTCGCC-3' |  |
| *ANG1* | F: 5'-CAGTCAGAGGCAGTACATGCT-3' | 98 bp |
|  | R: 5'-GCTTTTCATTTCCTATGTGGAATCTG-3' |  |
| *ANG2* | F: 5'-CGCTATGTGCTTAAAATACACC-3' | 196 bp |
|  | R: 5'-ATTTGTCGTTGTCTCCATCC-3' |  |
| *TIE1* | F: 5'-CACCCCAACATCATCAACCTCC-3' | 281 bp |
|  | R: 5'-TTCTCTCCGACCAGCACATTC-3' |  |
| *TIE2* | F: 5'-ACGCTTCACAACAGCTTC-3' | 283 bp |
|  | R: 5'-ATCTTCACTCCATTCCCCC-3' |  |
| *GAPDH* | F: 5'-TGCACCACCAACTGCTTAGC-3' | 87 bp |
|  | R: 5'-GGCATGGACTGTGGTCATGAG-3' |  |

**Table S2.** List of antibodies using western blot and immunohistochemistry

| Product | Cat. No. | Company |
| --- | --- | --- |
| Western blot | |  |
| LCN2 | AF1757 | R&D systems, Minneapolis, MN, USA |
| ECAD | 3195 | Cell signaling, Danvers, MA, USA |
| NCAD | 4061 | Cell signaling, Danvers, MA, USA |
| MMP1/8 | sc-137044 | Santa Cruz Biotech., Dallas, TX, USA |
| MMP2 | 4022 | Cell signaling, Danvers, MA, USA |
| MMP3 | sc-21732 | Santa Cruz Biotech., Dallas, TX, USA |
| MMP9 | 3852 | Cell signaling, Danvers, MA, USA |
| VIM | sc-6260 | Santa Cruz Biotech., Dallas, TX, USA |
| SNAI1 | sc-271977 | Santa Cruz Biotech., Dallas, TX, USA |
| ANG1 | sc-518157 | Santa Cruz Biotech., Dallas, TX, USA |
| TIE1 | sc-101587 | Santa Cruz Biotech., Dallas, TX, USA |
| TIE2 | sc-518076 | Santa Cruz Biotech., Dallas, TX, USA |
| GAPDH | 2118 | Cell signaling, Danvers, MA, USA |
| Rabbit IgG | ADI-SAB-300-J | Enzo Life Sciences, Farmingdale, NY, USA |
| Mouse IgG | ADI-SAB-100-J | Enzo Life Sciences, Farmingdale, NY, USA |
| Immunohistochemistry | |  |
| Ki-67 | sc-23900 | Santa Cruz Biotech., Dallas, TX, USA |
| CD31 | ab28364 | Abcam, Cambridge, MA, USA |
| LCN2 | ab23477 | Abcam, Cambridge, MA, USA |
| MMP3 | sc-21732 | Santa Cruz Biotech., Dallas, TX, USA |

**Table S3.** Serum and urine sample information used in ELISA

| **Variable** | | **Discovery** | **Validation 1** | **Validation 2** |
| --- | --- | --- | --- | --- |
| **BC patients** | N | 154 | 22 | 42 |
|  | Age (mean (SD)) | 72.3 (9.5) | 73.5 (9.7) | 71.4 (10.7) |
| Sex, N (%) | F | 23 (14.9) | 6 (27.3) | 8 (19.0) |
|  | M | 131 (85.1) | 16 (72.7) | 34 (81.0) |
| Type | NMIBC | 143 (92.9) | 20 (90.9) | 39 (92.9) |
|  | MIBC | 11 (7.1) | 2 (9.1) | 3 (7.1) |
|  | NA | 0 (0.0) | 0 (0.0) | 0 (0.0) |
| Grade | low | 44 (28.6) | 0 (0.0) | 2 (4.8) |
|  | high | 108 (70.1) | 20 (90.9) | 38 (90.5) |
|  | NA | 2 (1.3) | 2 (9.1) | 2 (4.8) |
| Stage | Tis | 14 (9.1) | 0 (0.0) | 5 (11.9) |
|  | Ta | 69 (44.8) | 5 (22.7) | 15 (35.7) |
|  | T1 | 51 (33.1) | 16 (72.7) | 18 (42.9) |
|  | ≥T2 | 11 (7.1) | 1 (4.5) | 3 (7.1) |
|  | NA | 9 (5.8) | 0 (0.0) | 1 (2.4) |
| **Controls** | N | 65 | 17 | 26 |
|  | Age (mean (SD)) | 62.5 (11.2) | 56.1 (12.8) | 57.6 (13.0) |
| Sex (%) | F | 32 (49.2) | 9 (52.9) | 13 (50.0) |
|  | M | 33 (50.8) | 8 (47.1) | 13 (50.0) |
| Classification | LUTS | 17 (26.2) | 0 (0.0) | 0 (0.0) |
|  | Urolithiasis | 1 (1.5) | 0 (0.0) | 0 (0.0) |
|  | Hematuria | 7 (10.8) | 0 (0.0) | 0 (0.0) |
|  | Urinary tract infection | 2 (3.1) | 0 (0.0) | 0 (0.0) |
|  | Other* | 17 (26.2) | 4 (23.5) | 5 (19.2) |
|  | Normal | 21 (32.3) | 13 (76.5) | 21 (80.8) |

**Abbreviations:** NMIBC, non-muscle invasive bladder cancer; MIBC: muscle invasive bladder cancer; LUTS: lower urinary tract symptoms; NA: not available

***Other:** Other non-malignant conditions
